# Potential of honey against the onset of autoimmune diabetes and its associated nephropathy, pancreatitis, and retinopathy in type 1 diabetic animal model

**DOI:** 10.1515/biol-2022-0039

**Published:** 2022-04-06

**Authors:** Sultan Fahad Al Nohair, Syed Suhail Ahmed, Mohamed Saleh Ismail, Ahdab Abdo El Maadawy, Manal A. Albatanony, Zafar Rasheed

**Affiliations:** Department of Family and Community Medicine, College of Medicine, Qassim University, Buraidah, Saudi Arabia; Department of Medical Microbiology, College of Medicine, Qassim University, Buraidah, Qassim, Saudi Arabia; Department of Nutrition and Food Sciences, Menoufia University, Shebin El-Kom, Egypt; Home Economics Dept, Faculty of Specific Education, Zagazig University, Zagazig, Egypt; Department of Family Medicine, College of Medicine, Qassim University, Unaizah, Saudi Arabia; Department of Medical Biochemistry, College of Medicine, Qassim University, P.O. Box 6655, Buraidah-51452, Saudi Arabia

**Keywords:** type 1 diabetes, diabetic rats, honey, blood sugar, lipid profile, histopathology

## Abstract

Honey has been used as a traditional remedy for various health benefits. This study investigated the potential of honey against the onset of autoimmune diabetes and its associated secondary complications in type 1 diabetic (T1D) experimental animals. Autoimmune diabetes was induced in Sprague Dawley rats, and at the same time, the rats were treated with honey or metformin. Sandwich ELISAs were used to estimate blood glucose, hemoglobin A1C (HbA1c), total cholesterol, and triglycerides. Histopathological examinations determined the T1D-induced lesions on kidneys, pancreas, cornea, and retina. Treatment of rats with honey during the course of T1D induction showed a significant reduction in fasting-blood-glucose and HbA1c (*p* < 0.01), and total lipid profile was also improved (*p* < 0.05). Not only these, but honey also reduced the T1D-induced lesions in the kidney, pancreas, and cornea/retina (*p* < 0.05). Metformin showed similar effects and was used as a positive control. In conclusion, honey showed therapeutic potential against the onset of autoimmune diabetes, as it reduces blood glucose/HbA1c and improves the lipid profile by reducing the plasma levels of total cholesterol, low-density lipoproteins (LDL), very low-density lipoprotein (VLDL), and triglycerides. Moreover, it also showed protective potential against the development of diabetic nephropathy, pancreatitis, and retinopathy.

## 1 Introduction

Diabetes mellitus (diabetes) is one of the major public health problems all over the globe, and its prevalence is continuously on the rise, and now it has threatened globally as an epidemic [1]. It is well documented that patients with uncontrollable diabetes have also been associated with several other major complications like retinopathy, renal impairment, cardiovascular disease, etc. These can further lead to an increase in the mortality rate worldwide [2]. Numerous pathogenic mechanisms are involved in the onset of diabetes, ranging from autoimmune destruction of the pancreatic β-cells with consequent insulin deficiency to abnormalities that result in resistance to insulin action [3]. It is reported that the metabolic abnormalities in carbohydrate, fat, and protein in diabetes could be due to the lack of supply of insulin, which results from inadequate insulin secretion and/or diminished tissue responses to insulin at one or more points in the complex pathways of hormone action. Impairment of insulin secretion and defects in insulin action frequently coexist, which may be the primary cause of hyperglycemia [4,5]. Polyuria, polydipsia, weight loss, polyphagia, and blurred eyes are the symptoms of chronic diabetes [6]. Moreover, abnormalities associated with growth and susceptibility to certain bacterial, fungal, and viral infections may also be accompanied by diabetes [7]. Long-term complications of chronic hyperglycemia include retinopathy with the potential loss of vision; nephropathy leading to renal failure; peripheral neuropathy with risk of foot ulcers, amputations, and Charcot joints; and autonomic neuropathy causing gastrointestinal, genitourinary, and cardiovascular symptoms and sexual dysfunction [8,9]. Furthermore, diabetic patients have an increased incidence of atherosclerotic cardiovascular, peripheral arterial, and cerebrovascular disease [10]. Not only these, but diabetic patients are also generally hypertensive, and lipoprotein metabolic abnormalities are often reported in them [11,12]. It is now well-known that diabetes has a multifactorial etiology that includes genetic, social, and environmental factors [2,12,13,14]. Till now, no medication is considered curable for diabetes, but the use of alternative medicines is somewhat useful for controlling the sugar levels in these patients [15]. Few studies at cellular and animal models reported that honey ameliorates hyperglycemia and reduces the levels of oxidative damage under diabetic conditions [16,17]. Besides, honey was also reported to increase high-density lipoprotein (HDL) and protect the liver by reducing hepatic transaminases [18,19]. However, controversial reports were also published for diabetic patients, mentioning that honey has increased the levels of HbA1c [20]. Most importantly, studies also reported that the combination of honey with anti-diabetic drugs improved the levels of body antioxidants status and helped normalize the hyperglycemic conditions and reduced abdominal pain, and was helpful for wounds, asthma, and burns [19,20,21]. In support of these, studies also showed that honey has the benefit of managing diabetes through the control of hyperglycemia and limiting other metabolic disorders [22,23]. In spite of these benefits of honey, it is still not clear whether or not honey provides anti-glycemic benefits to patients during the development of type 1 diabetes (T1D). In view of these, this study was hypothesized for the first time that the administration of royal jelly honey prevents the onset of T1D. The honey was tested against the onset of T1D in a rat model of TID to investigate this hypothesis. Our novel findings showed that the royal jelly honey reduces blood sugar levels by reducing the blood glucose or HbA1c and improves the lipid profiles during the onset of T1D in experimental rats. Furthermore, the royal jelly honey also reduces the T1D-induced lesions on the kidney, pancreas, cornea, and retina, indicating that it is also helpful to prevent the onset of diabetes-associated secondary complications.

## 2 Methods

### 2.1 Study setting, duration, and design

This is an animal-based experimental study performed in the College of Medicine, Qassim University, KSA, from Jan 2019 to Aug 2019. The study was designed to determine the specific role of royal jelly honey in T1D animal model.

### 2.2 Acclimatization of experimental rats

A total of 50 Sprague-Dawley rats (aged 4 weeks; weighing 120–130 g) were collected from the Animal Unit of the Faculty of Pharmacy, King Saud University, Saudi Arabia. The rats were housed in plastic cages at a controlled temperature of 22  ± 3°C with a 12 h light/dark cycle for 2 weeks in the College of Medicine, Qassim University, Saudi Arabia. Animals were fed with standard chow diet, comprised of crude protein 20.0%, crude fat 4.0%, crude fiber 3.5%, ash 6.0%, salt 0.5%, calcium 1.0%, phosphorus 0.6%, vitamin A 20.0 IU/g, vitamin D 2.2 IU/g, vitamin E 70.0 IU/kg, energy 2,850 ME kcal/kg, and trace minerals such as cobalt, copper, iodine, iron, manganese, selenium, zinc (catalog # F-1005, The Saudi Grains Organization, SAGO, Saudi Arabia), and the diet was given as described previously [24].


**Ethical approval:** The research related to animal use has been complied with all the relevant national regulations and institutional policies for the care and use of animals, and has been approved by the Institutional Review Board of Deanship of Scientific Research, Qassim University (IRB approval #19-09-02).

### 2.3 Induction and treatment of T1D rats

After 2 weeks of acclimatization of rats, T1D was induced by intraperitoneal injections of streptozotocin (STZ) as described previously [25]. Briefly, T1D was induced in Sprague Dawley rats by intraperitoneal injections with STZ (75 mg/kg; catalog # S0130, Sigma-Aldrich, St. Louis, MO, USA) dissolved in a citrate buffer (0.1 mol/L sodium citrate and 0.1 mol/L citric acid, and pH 4.5), and at the same time, the rats were also administered with royal jelly honey (0.3–0.9 mL/kg; Buram GmbH, Berlin, Germany) or metformin (CAS # 1115-70-4, Calbiochem). The final treatment dose of royal jelly honey was decided to be 0.85 mL/kg after standardization as this dose showed the best inhibitory effect of T1D in STZ -treated rats. Whereas a standardized dose of metformin 70 mg/kg was given to the diabetic rats as described previously [26]. All the experimental rats were divided into 4 groups (*n* = 40). The first group was untreated healthy rats used as controls (*n* = 10), the second group was T1D rats (*n* = 10; 75 mg/kg STZ), the third group was treated with honey (*n* = 10; 0.85 mL/kg) during the course of T1D induction, and the fourth group was treated with metformin (*n* = 10; 80 mg/kg) during the course of T1D induction.

### 2.4 Estimation of diabetic markers in blood samples of experimental rats

The fasting blood samples were collected after 4 weeks of continuous T1D induction and treatment with honey or metformin. Whole blood was used for HbA1c assay, and plasma samples were used to estimate blood glucose, total cholesterol, low-density lipoproteins (LDL), very low-density lipoprotein (VLDL), HDL, and triglycerides using the sandwich ELISAs. The levels of blood HbA1c were measured by rat-specific glycated hemoglobin A1c ELISA Kit (catalog # MBS2033689, MyBioSource Inc., San Diego, CA, USA), whereas the fasting plasma glucose was measured using rat specific glucose ELISA kit (catalog # MBS7233226, MyBioSource Inc.). The total cholesterol was measured using rat total cholesterol ELISA Kit (#MBS775433, MyBioSource Inc.), the levels of HDL in the plasma was measured by the rat specific high-density lipoprotein ELISA Kit (catalog #MBS704516, MyBioSource Inc.), VLDL was measured by rat very low-density lipoproteins, ELISA Kit (#MBS026726, MyBioSource Inc.), LDL was measured by rat low-density lipoprotein ELISA Kit (#MBS702165, MyBioSource Inc.) and the levels of triglycerides in experimental rats were measured by the rat triglyceride ELISA Kit (catalog # MBS726298, MyBioSource Inc.) as instructed by the manufacturer’s instructions (MyBioSource Inc., San Diego, CA, USA).

### 2.5 Histological examination

Samples of kidneys, pancreas, eye cornea, and retina were taken from the experimental rats, fixed in neutral buffered formalin for 48 h, and processed for hematoxylin-eosin histopathological examination using routine methods as described previously [27]. Briefly, the collected tissues of kidneys, pancreas, eye cornea, and eye retina from rats were immersed in 4% paraformaldehyde for 48 h and then dehydrated step by step using a gradient of ethanol (50, 70, 80, 90, 95, and 100%). Tissue samples were immersed in xylene for 30 min and incubated in paraffin at 65°C overnight. Once embedded in wax, the samples were cut serially into 5 μm thick sections using a microtome (Leica Biosystem, Wetzlar, Germany) and spread over microscopy slides. The sections were deparaffinized with fresh xylene for 10 min, rehydrated with a gradient of ethanol (100, 90, 80, and 70%), and then washed three times with deionized double-filtered distilled water. The sections were analyzed using hematoxylin-eosin staining and examined with a light microscope digital camera (Nikon Instruments, Tokyo, Japan).

### 2.6 Statistical analysis

The data were presented as mean value ± SD and were statistically compared for the determination of levels of significance between the experimental rat groups by One-Way ANOVA followed by Tukey’s *post hoc* analysis using GraphPad Prism-8 (San Diego, CA, USA). The *p* < 0.05 was considered statistically significant.

## 3 Results

### 3.1 Effect of honey on blood glucose and HbA1C levels during the onset of T1D

**Figure 1:**
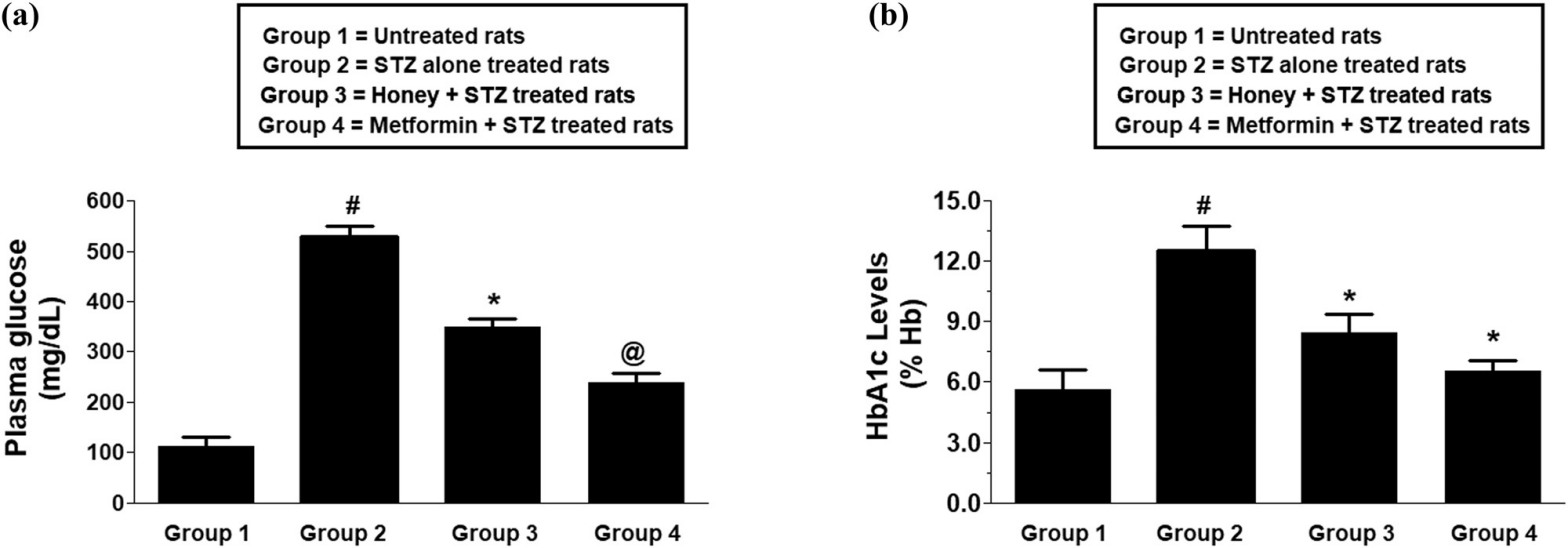
Effect of honey on T1D-induced diabetic markers in experimental animals. Fasting plasma glucose levels (a) and blood HbA1c levels (b) in control and experimental rats. Data shown are mean value ± SD of 10 independent experimental groups. ^#^ *p* < 0.001 vs group 1 rats; ^*^ *p* < 0.01 vs group 2 rats; ^@^ *p* < 0.001 vs group 2 rats. Abbreviations: T1D, type 1 diabetes; HbA1c, glycosylated hemoglobin A1c; STZ, streptozotocin.

The effect of honey on the normalization of fasting blood glucose levels on the rats during the onset of T1D was evaluated. Fasting plasma glucose levels were analyzed in all four experimental rat groups. The average fasting plasma glucose levels (± SD) of 10 independent assays for the group 1 rats (untreated rats), group 2 (STZ alone treated rats or T1D rats group), and group 3 (honey + STZ treated rats group or honey-treated T1D group) were found to be 112.0 ± 18.2, 530.0 ± 19.2, and 349.1 ± 17.5 mg/gL, respectively (Figure 1a). The data clearly showed that the treatment of honey during the onset of T1D significantly reduces the fasting plasma glucose levels (*p* < 0.05). Whereas the treatment of T1D rats with metformin (group 4) also showed a significant reduction of fasting plasma glucose levels (*p* < 0.01) and was used as a positive control (Figure 1a). In the same way, the percent HbA1c levels were analyzed in the same studied experimental rat groups. The average percent HbA1c levels (±SD) of 10 independent assays for the control group, T1D group, and honey-treated T1D group were found to be 5.6 ± 0.9, 12.6 ± 1.2, and 8.4 ± 0.9 mg/gL, respectively (Figure 1b). The data showed that the administration of honey during the onset of T1D significantly reduced the precent HbA1c levels (*p* < 0.05), whereas metformin also showed similar results and was used as a positive control (Figure 1b).

### 3.2 Effect of honey on lipid profile during the onset of T1D

**Figure 2:**
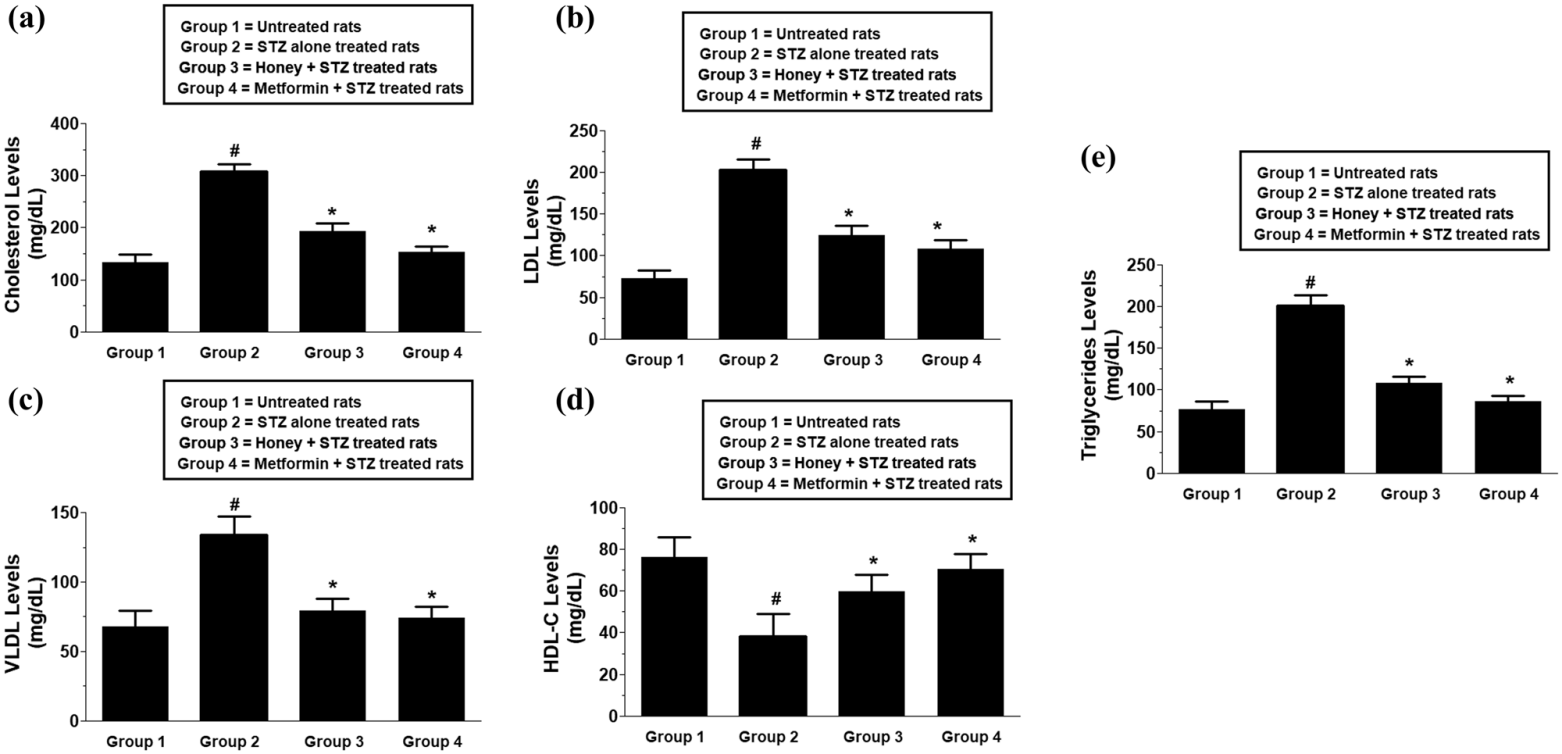
Effect of honey on T1D-induced lipid profile in experimental animals. Total cholesterol levels (a), LDL levels (b), VLDL levels (c), HDL levels (d), and triglyceride levels (e) in the plasma of control and experimental rats. Data shown are mean value ± SD of 10 independent experimental groups. ^#^ *p* < 0.01 vs group 1 rats; ^*^ *p* < 0.05 vs group 2 rats. Abbreviations: T1D, type 1 diabetes; LDL, low-density lipoprotein-cholesterol; VLDL, very low-density lipoprotein-cholesterol; HDL, high-density lipoprotein-cholesterol; STZ, streptozotocin.

The effect of honey on the lipid profile was analyzed in all four experimental rat groups by estimating the plasma levels of total cholesterol, LDL levels, VLDL levels, HDL levels, and triglycerides (Figure 2). The average total cholesterol levels (±SD) of 10 independent assays for the control group, T1D group, honey-treated T1D group, and metformin-treated T1D rats were found to be 132.4 ± 15.3, 308.2 ± 13.6, 193.1 ± 15.8, and 153.1 ± 10.5 mg/gL, respectively (Figure 2a). The data clearly showed that the treatment of honey during the onset of T1D significantly reduced the total cholesterol levels (*p* < 0.01). Whereas the treatment of T1D rats with metformin also showed a significant reduction in total cholesterol levels (*p* < 0.01) and was used as a positive control of the experiments (Figure 2a). Similarly, the levels of LDL were also reduced by the treatment of honey or metformin during the onset of T1D. The average LDL levels (±SD) of 10 independent assays for the control group, T1D group, honey-treated T1D group, and metformin-treated T1D group were found to be 72.4 ± 9.5, 203.2 ± 12.6, 124.0 ± 11.8 and 108.1 ± 10.5 mg/gL, respectively (Figure 2b). To determine the data in more depth, the levels of VLDL were also analyzed and were found to be significantly reduced in the rat group treated with honey or metformin (Figure 2c). The average VLDL levels (±SD) of 10 independent assays for the control group, T1D group, honey-treated T1D group, and metformin-treated T1D group were found to be 45.3 ± 7.5, 89.2 ± 8.8, 52.7 ± 5.8, and 49.3 ± 5.3 mg/gL, respectively (Figure 2c). In contrast to these, HDL levels in all 4 experimental groups showed inverse results. The average HDL levels (±SD) of 10 independent assays for the control group, T1D group, honey-treated T1D group, and metformin-treated T1D group were found to be 76.3 ± 9.5, 38.5 ± 10.7, 59.6 ± 8.4, and 70.5 ± 7.3 mg/gL, respectively (Figure 2d). The data showed that T1D induction reduced HDL levels, whereas the treatment of rats with honey or metformin significantly increased HDL levels in the plasma of these experimental rats (*p* < 0.05). To further validate these results, the plasma levels of triglycerides were analyzed. The average triglycerides levels (±SD) of 10 independent assays for the control group, T1D group, honey-treated T1D group, and metformin-treated T1D group were calculated to be 75.4 ± 9.6, 201.2 ± 12.7, 107.7 ± 8.3, and 85.7 ± 7.4 mg/gL, respectively (Figure 2e). The data showed that T1D induction in experimental rats significantly increased the levels of triglycerides (*p* < 0.001), whereas the treatment of rats with honey or metformin significantly decreased the levels of triglycerides during the onset of this autoimmune diabetes (*p* < 0.01).

### 3.3 Effect of honey on T1D-induced histopathological changes in kidneys during the onset of T1D in experimental rats

**Figure 3:**
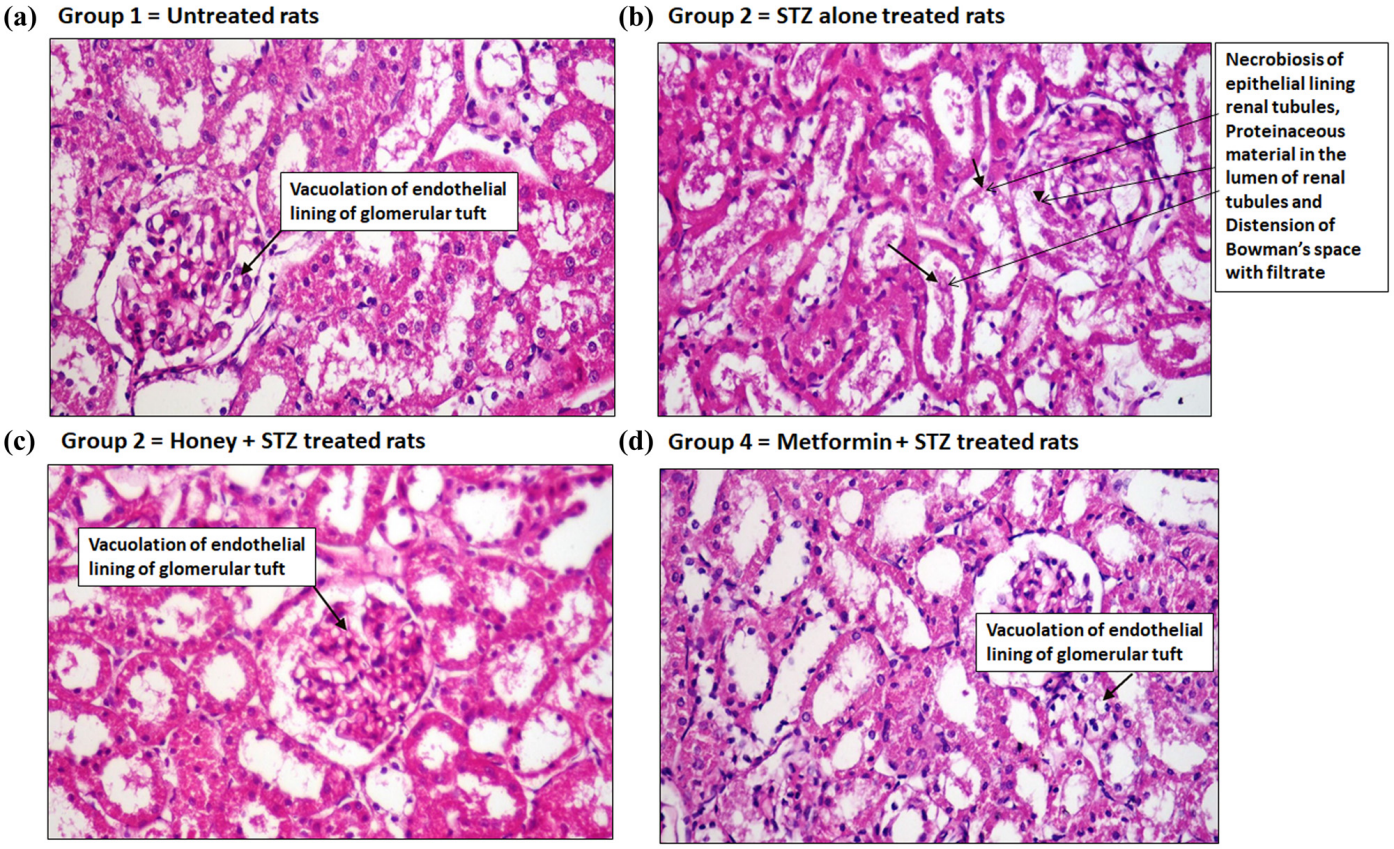
Hematoxylin-eosin examinations of kidney sections. (a) Kidney section of a rat from normal group (group 1) showing vacuolation of endothelial lining glomerular tuft. (b) Kidney section of a rat from T1D group (group 2) showing necrobiosis of epithelial lining renal tubules, proteinaceous material in the lumen of renal tubules, and distension of Bowman’s space with filtrate. (c) Kidney section of a rat from honey-treated T1D group (group 3) showing vacuolation of endothelial lining glomerular tuft. (d) Kidney section of a rat from metformin-treated T1D group (group 4) showing vacuolation of epithelial lining some renal tubules. All kidney sections were processed through hematoxylin-eosin histopathological examination (H&E ×400).

Hematoxylin-eosin histopathological examination showed that the kidney sections of rats from control group 1 revealed no changes except vacuolation of endothelial lining glomerular tuft (Figure 3a). Whereas the kidney sections from group 2 diabetic rats revealed necrobiosis of epithelial lining renal tubules, proteinaceous material in the lumen of renal tubules, and distension of Bowman’s space with filtrate (Figure 3b). Interestingly, the kidney sections obtained from the honey-treated group 3 rats showed vacuolation of endothelial lining glomerular tuft (Figure 3c). Similarly, the group 4 rats treated with metformin also showed vacuolation of epithelial lining some renal tubules (Figure 3d).

### 3.4 Effect of honey on T1D-induced histopathological changes in pancreatic sections during the onset of T1D in experimental rats

**Figure 4:**
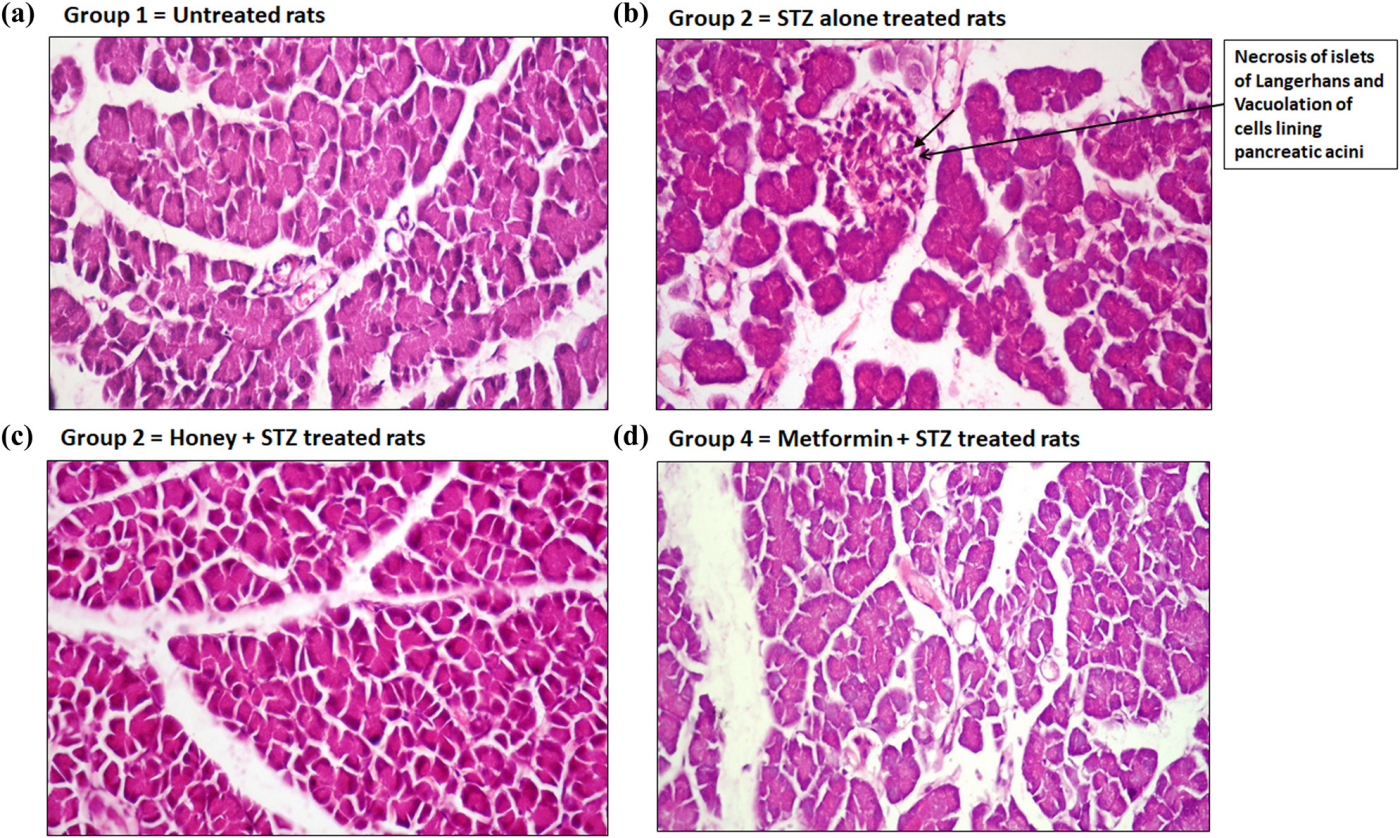
Hematoxylin-eosin examinations of pancreatic sections. (a) Pancreatic section of a rat from normal group (group 1) showed no histopathological changes. (b) Pancreatic section of a rat from T1D group (group 2) showed necrosis of cells of islets of Langerhans and vacuolation of cells lining pancreatic acini. (c) Pancreatic section of a rat from honey-treated T1D group (group 3) showed no major histopathological changes. (d) Pancreatic section of a rat from metformin-treated T1D group (group 4) showed no major histopathological changes. All pancreatic sections were processed through hematoxylin-eosin histopathological examination (H&E ×400).

Pancreatic sections of rats from group 1 normal rats revealed no histopathological changes (Figure 4a). On the other hand, pancreatic sections from T1D rats of group 2 showed necrosis of cells of islets of Langerhans and vacuolation of cells lining pancreatic acini (Figure 4b). Importantly, the administration of honey to T1D rats of group 3 showed no adverse histopathological modifications (Figure 4c), and metformin also normalized the T1D-induced pancreatic histopathological alterations as observed in group 4 rats (Figure 4d).

### 3.5 Effect of honey on T1D-induced histopathological changes in corneal eye sections during the onset of T1D in experimental rats

**Figure 5:**
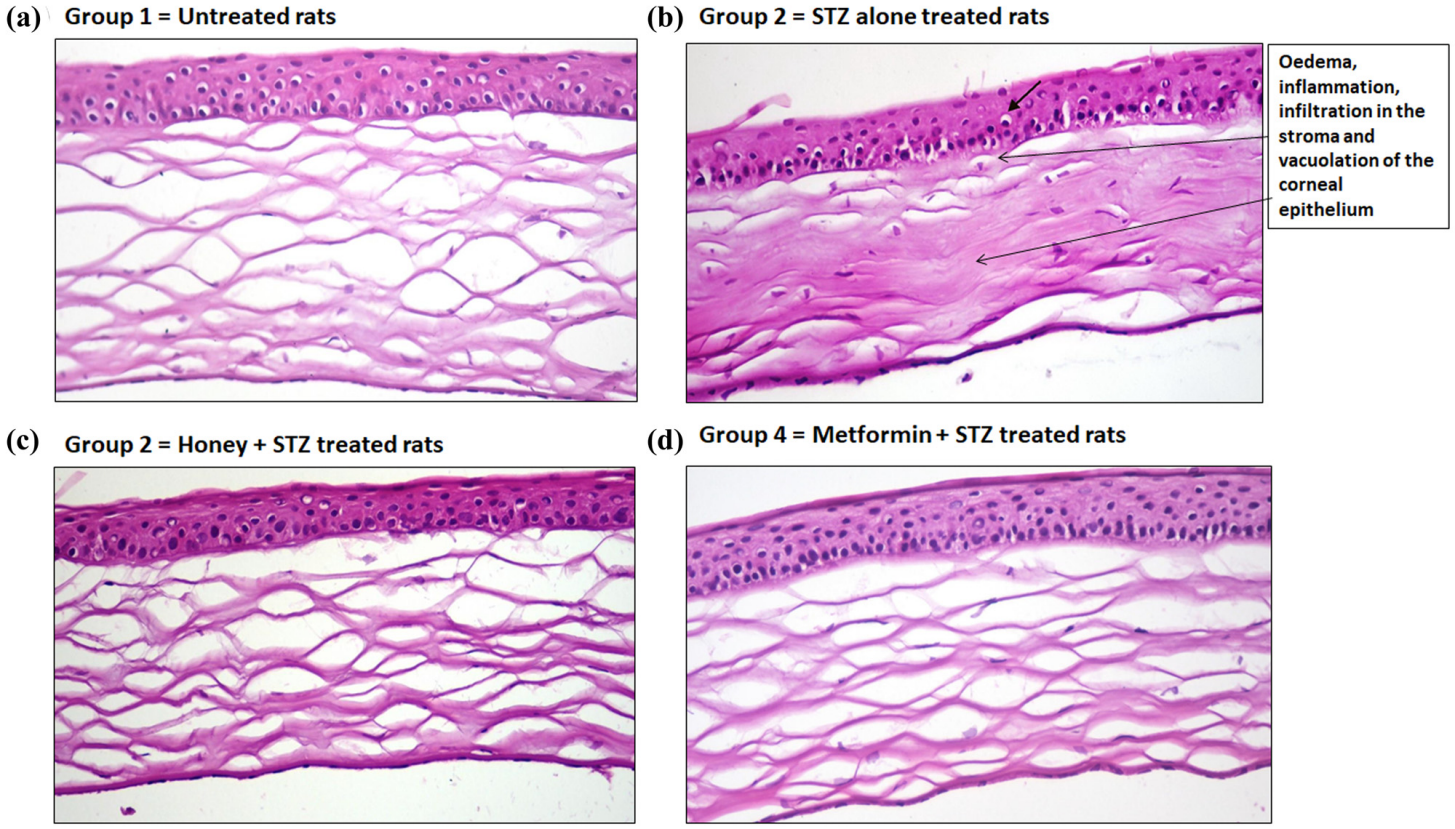
Hematoxylin-eosin examinations of eye corneal sections. (a) Corneal section of a rat from normal group (group 1) showed no histopathological changes. (b) Corneal section of a rat from T1D group (group 2) showed edema, inflammatory cells infiltration in the stroma, and vacuolation of corneal epithelium. (c) Corneal section of a rat from honey-treated T1D group (group 3) showed no major histopathological changes. (d) Corneal section of a rat from metformin-treated T1D group (group 4) showed no major histopathological changes. All corneal sections were processed through hematoxylin-eosin histopathological examination (H&E ×400).

Corneal sections from untreated rats revealed no histopathological changes (Figure 5a). On the other hand, corneal sections from the T1D group 2 rats revealed edema and inflammatory cells infiltration in the stroma and vacuolation of the corneal epithelium (Figure 5b). Notably, the administration of honey to T1D rats showed no adverse histopathological modifications (Figure 5c), and metformin also normalized the T1D-induced pancreatic histopathological alterations (Figure 5d).

### 3.6 Effect of honey on T1D-induced histopathological changes on eye retinal sections during the onset of T1D in experimental rats

**Figure 6:**
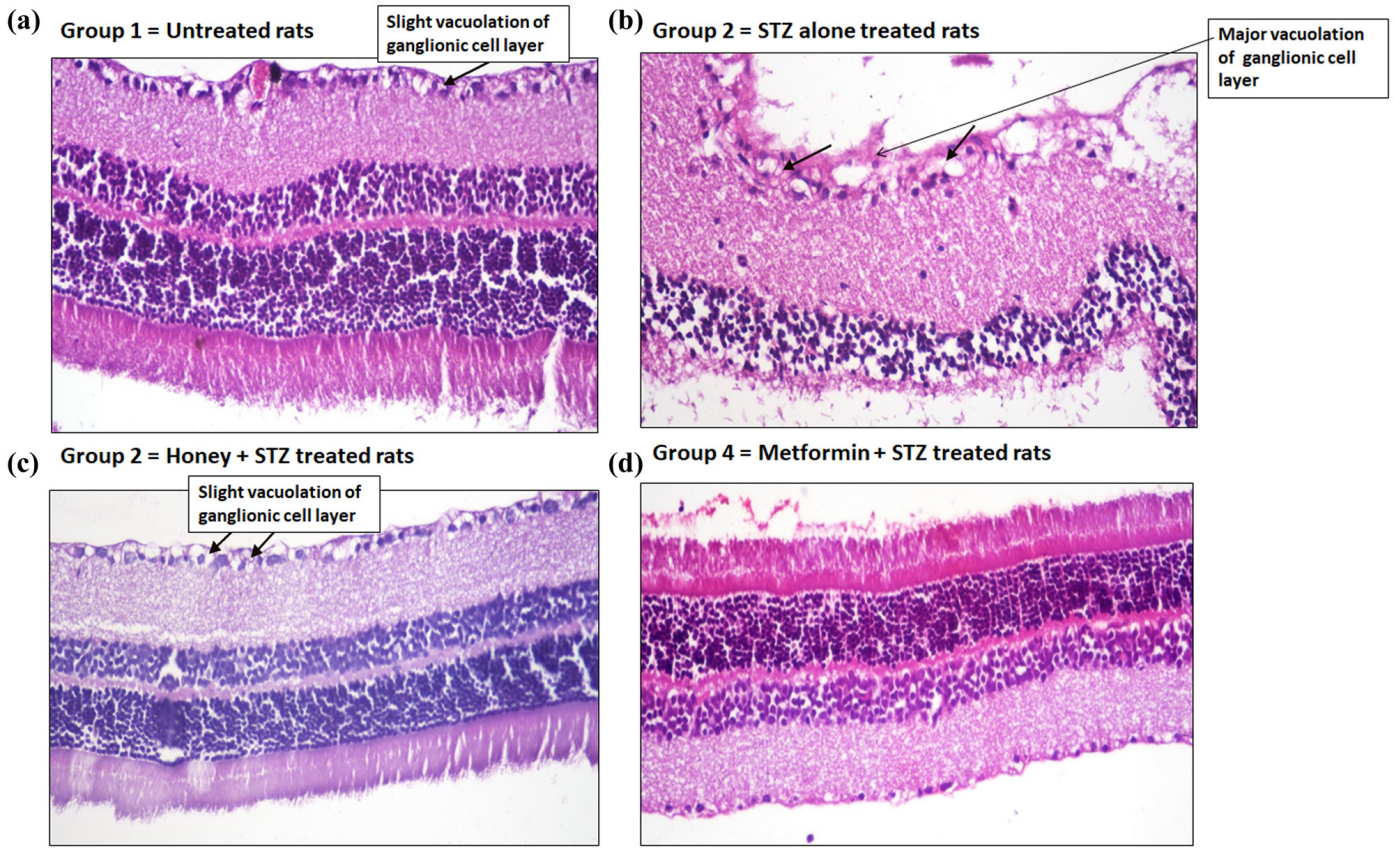
Hematoxylin-eosin examinations of eye retinal sections. (a) Retinal section of a rat from normal group (group 1) showed slight vacuolation of ganglionic cell layer. (b) Retinal section of a rat from T1D group (group 2) showed vacuolation of ganglionic cell layer. (c) Retinal section of a rat from honey-treated T1D group (group 3) showed slight vacuolation of ganglionic cell layer. (d) Retinal section of a rat from metformin-treated T1D group (group 4) showed no major histopathological changes. All retinal sections were processed through hematoxylin-eosin histopathological examination (H&E ×400).

Retinal sections of normal rats in group 1 revealed no histopathological changes except slight vacuolation of the ganglionic cell layer (Figure 6a), whereas the retinal sections from T1D rats group 2 showed major vacuolation of ganglionic cell layer (Figure 6b). Importantly, treatment with honey to T1D rats normalized these histopathological changes, except slight vacuolation of ganglionic cell layers was noticed (Figure 6c), and metformin also showed normalization of the T1D-induced retinal alterations (Figure 6d).

## 4 Discussion

To the best of our knowledge, this is the first study that shows that honey reduces the levels of blood glucose and HbA1c and also improves the lipid profile during the onset of autoimmune diabetes. Natural dietary products and their bioactive ingredients are widely used as alternative medicines for the treatment of numerous human disorders all over the globe [28,29,30,31,32,33,34,35,36], but studies are still limited to proving their therapeutic efficacies. Similarly, the health benefits of royal jelly have also been well documented as it is widely used as a healthy food source and currently being used in the manufacturing of several commercial medical products, foods, and cosmetics all over the world, but its role as a therapeutic agent is still largely unknown [37,38,39,40]. Several published reports showed that royal jelly contains numerous bioactive components, including bioactive fatty acids, proteins, and phenolic compounds. These are reported to have antimicrobial activity, anti-inflammatory activity, antioxidant activity, and it is also reported that they possess cholesterol-reducing activity, vasodilative and hypotensive activities, and activity against tumor formation [37,38,39,40,41]. In this study, we used royal jelly honey administration to the rats during the onset of autoimmune diabetes. This royal jelly honey was reported to contain a high amount of fructose [38,42]. Previous studies have shown that dietary fructose is easily absorbed in the gastrointestinal tract and reported to reduce the blood glucose in patients with both type 1 and type 2 diabetes [42]. It is also reported that fructose metabolism is largely independent of insulin, and it is quickly removed by the liver, therefore several investigators suggested that its ingestion does not increase the blood sugar, due to these reasons it is widely used as a sweetener by patients with diabetes all over the world [42]. In the present study, the effect of royal jelly honey on the normalization of fasting blood glucose levels was tested on the rats developing T1D. Our data clearly points out that the treatment with royal jelly honey during the development of T1D significantly reduced the fasting plasma glucose levels and blood HbA1c. Our findings also showed that the treatment of rats with royal jelly honey during the development of T1D also significantly improved the lipid profile by reducing the blood levels of total cholesterol, triglycerides, LDL, VLDL, and upregulating the blood levels of HDL, clearly indicating the potential role of royal jelly honey against the onset of T1D. These novel findings are well supported by the previous studies performed on natural honey as it was also found to have several health benefits against bacterial infection, oxidative stress, hepatotoxicity, hypertension, and also reported to improve the reproductive system [43,44].

For the last two decades, it is clear that the onset of T1D has also been associated with several secondary complications such as nephropathy, retinopathy, and complications with pancreatic dysfunctions that considerably increase global morbidity and mortality [45,46,47]. In this regard, we further investigated the protective potential of the royal jelly honey against the T1D-induced lesion in the kidney, pancreas, cornea, and retina in experimental rats during the development of T1D-induced secondary complications. Our histopathological examination of kidney sections showed that T1D-induced lesions like necrobiosis of epithelial lining on renal tubules, proteinaceous material in the lumen of renal tubules, and distension of Bowman’s space with filtrate have been significantly healed upon treatment with royal jelly honey, indicating that royal jelly honey provides the protection against diabetic nephropathy. Similarly, our histological findings on pancreatic sections showed that the treatment of rats with royal jelly honey prevents T1D-induced necrosis of islets of Langerhans and vacuolation of cells lining pancreatic acini, clearly suggesting that royal jelly honey also provides protection against the development of pancreatitis. To study the effect of royal jelly honey in more detail, the histopathological examination of rat eye corneal sections and retinal sections were analyzed. Our novel findings revealed that treatment with royal jelly honey inhibited the T1D-induced edema and inflammation in the stroma and inhibited the T1D-induced vacuolation of corneal epithelium. Similarly, the royal jelly honey treatment also decreased the levels of major vacuolation of ganglionic cell layers in the retina. These findings point out that royal jelly honey also provides protection against the onset of diabetic retinopathy. Taken together, these novel findings not only suggested that the royal jelly honey reduced the blood glucose or HbA1c levels but also improved the lipid profile during the development of autoimmune diabetes. Furthermore, it also provides protection against the induction of diabetic-associated secondary complications. This study has a few limitations. The first and the obvious limitation was only one type of honey was tested, and the study was conducted on a single breed of rats. Therefore, we recommend other studies that will test the other types of honey and will involve multiple breeds of rats.

## 5 Conclusion

This is the first study to determine the protective potential of royal jelly honey during the development of autoimmune diabetes and its associated secondary complications in experimental animals. The novel findings showed the potential of royal jelly honey in the prevention of the onset of T1D by reducing the levels of blood sugar or HbA1c and improving the lipid profile. Moreover, the data also concluded that royal jelly honey also provides protection against the development of diabetic nephropathy, pancreatitis, and retinopathy.
